# MRI features of primary cardiac lymphoma: case report and literature review

**DOI:** 10.3389/fonc.2025.1596237

**Published:** 2025-07-04

**Authors:** Dongmei Xie, Yudan Li, Chunyong Tang, Qing Zou

**Affiliations:** Department of Radiology, People’s Hospital of Deyang City, Deyang, Sichuan, China

**Keywords:** primary cardiac lymphoma, MRI, right atrial, tumor, literature review

## Abstract

**Purpose:**

This study aimed to further enhance the understanding of the imaging features of primary cardiac lymphoma (PCL), improve preoperative diagnostic accuracy, and reduce the misdiagnosis rates.

**Methods:**

This study reports on a case of a 63-year-old man who presented with 3-day-long dizziness and bilateral lower limb edema. Initial transthoracic echocardiography detected a 4.5-cm × 5-cm mass without blood flow in the right atrium, suspected as a thrombus or tumor. Subsequently, cardiac MRI (3.0T Ingenia, Philips, Best Netherlands) was performed. Pre-contrast black blood turbo spin echo (TSE) T1-weighted imaging (T1WI), T2-weighted imaging (T2WI), T2 fat saturation (STIR), and retrospective ECG-triggered balanced turbo field echo (steady-state free precession) sequences on short and long axes were used.

**Results:**

MRI showed a roundish solid mass in the right atrium with clear margins, broad-based attachment to the anterior/posterior walls, adjacent right atrial wall thickening, inferior vena cava inlet narrowing, and protrusion into the tricuspid orifice during atrial systole. First-pass perfusion presented homogeneous progressive enhancement, while the delayed phase showed patchy irregular enhancement. Due to limited knowledge about PCL, a preliminary diagnosis of cardiac myxoma was made. Eventually, tumor resection was carried out, and postoperative pathology confirmed it as right atrial diffuse large B-cell lymphoma (DLBCL) with a Ki-67 proliferation index of 90%. Although the symptoms improved post-surgery, the patient refused further chemotherapy and died shortly.

**Conclusion:**

This case highlights that MRI plays a significant role in the diagnosis of PCL, helping to identify characteristic imaging features and reduce misdiagnosis. Preoperative biopsy is crucial for accurate diagnosis, and chemotherapy is essential for improving patient survival. Further research is needed to better understand the imaging features of this rare tumor and optimize treatment strategies.

## Introduction

Primary cardiac lymphoma (PCL) is a rare neoplasm involving the heart, the pericardium, or both. The lack of typical manifestations makes it difficult to diagnose at an early stage that can be discovered in the past. PCL refers to non-Hodgkin lymphoma that involves the heart, pericardium, or both. Lymphoma that is mainly located in the heart, which is one of the extremely rare tumors, accounts for only 1% of primary cardiac tumors and 0.5% of extranodal lymphomas (1). Diffuse large B-cell lymphoma is the most common and frequently involves the right atrium (RA) and the right ventricle (2). It often occurs after the age of 50 (3). Early diagnosis is difficult due to the lack of typical imaging manifestations, particularly magnetic resonance manifestations of PCL, which is usually determined using echocardiography in previous examinations. This article describes a rare case of PCL in the right atrium. To the best of our knowledge, only a few similar cases have been reported.

## Case presentation (symptom onset→initial echocardiography→MRI→surgery→follow-up)

The patient, a 63-year-old man, presented to Deyang People’s Hospital with 3-day-long progressive dizziness and lower limb edema.

### Initial examination

**Figure 1:**
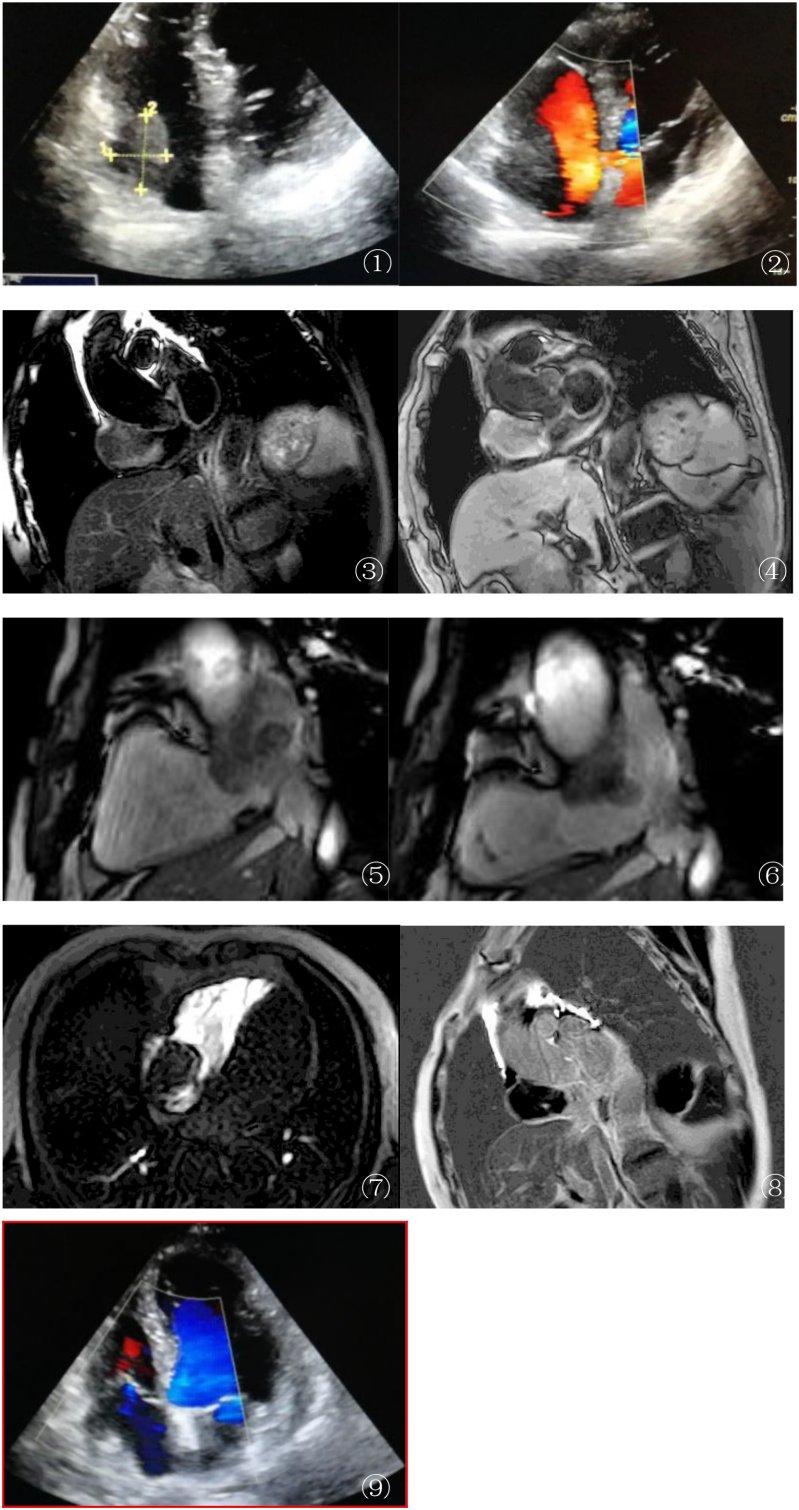
**(A, B)** Echocardiography findings: Transthoracic echocardiography **(A)** detected a 4.5-cm × 5-cm mass in the right atrium (RA). Color Doppler ultrasound **(B)** revealed no intralesional vascular flow, raising suspicion of either a thrombus or an avascular tumor. These initial findings lacked specificity for definitive diagnosis, highlighting the need for further imaging. **(C, D)** MRI anatomical imaging: Axial T2-weighted single-shot turbo spin echo (T2W–SSH–TSE) **(C)** and T1-weighted turbo spin echo (T1W–TSE–SA) **(D)** sequences demonstrated an isointense mass in the RA with broad-based attachment to the anterior and posterior walls. Adjacent structural changes were notable: the RA wall exhibited thickening and edema, and the inferior vena cava (IVC) inlet showed stenosis, indicating potential local invasion. **(E, F)** Cardiovascular magnetic resonance (CMR) cine sequence (balanced turbo field echo, BTFE–BH–LA): During atrial systole **(E)** (*left panel*), the mass protruded into the tricuspid orifice, causing secondary tricuspid regurgitation. In diastole **(F)** (*right panel*), the mass retracted back into the RA, closely adjacent to the superior vena cava (SVC) and IVC orifices. This dynamic motion pattern provided insights into the mechanical impact of the mass on cardiac hemodynamics. **(G)** First-pass perfusion MRI: Unusually, the mass showed no enhancement during the arterial phase of first-pass perfusion **(F)**, contrasting with the typical hypervascularity observed in diffuse large B-cell lymphoma (DLBCL). This atypical feature suggested unique tumor microvascular characteristics or potential sampling bias, warranting further investigation. **(H)** Late gadolinium enhancement (LGE) MRI: Axial images revealed patchy, irregular enhancement of the mass, with non-enhancing central foci consistent with tumor necrosis. Notably, the edematous atrial wall showed no abnormal enhancement, helping to differentiate the tumor tissue from reactive changes. **(I)** During the re-examination 2 months after surgery, the patient was found to have cardiac enlargement and pericardial effusion, with no recurrence in the surgical area.

On admission, transthoracic echocardiography found a 4.5-cm × 5-cm space-occupying lesion in the right atrial chamber (see **Figure 1A**). Color Doppler showed no intralesional blood flow (**Figure 1B**), raising the suspicion of a thrombus or tumor.

### Laboratory and physical examination

During hospitalization, the laboratory results of the patient were as follows: cardiac troponin T (cTNT), 0.022 ng/ml; B-type natriuretic peptide (NT-proBNP), 3,022 pg/ml; creatine kinase (CK), 82 U/L; creatine kinase–myocardial band (CK-MB), 15 U/L; serum creatinine (SCr), 74 μmol/L; uric acid (UA), 433 μmol/L; Na^+^, 138 mmol/L; K^+^, 4.7 mmol/L;Ca^2+^, 2.14 mmol/L; fasting blood glucose (FBG), 5.5 mmol/L; HbA1c, 6.5%; hemoglobin (Hb), 125 g/L; red blood cell (RBC), 5.83 × 10^9^/L; D-dimer, 0.65 g/L; and erythrocyte sedimentation rate (ESR), 19 mm/h. The viral antibody screening and tumor markers were negative. Physical examination revealed no jugular vein distension. The thoracic cage was symmetrical, the intercostal spaces were normal, the bilateral respiratory movements were consistent, and tactile fremitus was symmetrical. The breath sounds in both lungs are clear, with a few fine moist rales in the lower lobes of both lungs. The precordial region had no prominence, the cardiac dullness border was not enlarged, heart rhythm was irregular, no murmur was heard at the apex of the heart, and no thrill was detected. No enlargement of the superficial lymph nodes was detected.

### MRI examination

To further evaluate the mass, cardiac magnetic resonance imaging (MRI) (3.0T Ingenia, Philips, Best Netherlands) was performed. The imaging protocol included pre-contrast black blood turbo spin echo T1-weighted imaging (T1WI), T2-weighted imaging (T2WI), T2 fat saturation (short-tau inversion recovery, STIR), and retrospective ECG-triggered balanced turbo field echo (steady-state free precession, SSFP) sequences. The sequences were oriented on the short and long axes (two-, three-, and four-chamber views of the heart). MRI showed a roundish mass in the RA with clear margins and broad-based attachment to the anterior/posterior walls. Adjacent RA wall thickening and edema, as well as narrowing of the inferior vena cava (IVC) inlet, were observed (**Figures 1C–F**). The SSFP sequence showed that the mass protruded into the tricuspid orifice during atrial systole (causing secondary tricuspid regurgitation) and returned to the RA during diastole. First-pass perfusion demonstrated homogeneous progressive enhancement of the mass, while the late gadolinium enhancement (LGE) showed patchy irregular enhancement (**Figures 1G, H**). To better highlight the key features in the figures, arrows and brief descriptions have been added to indicate the location of the mass, wall thickening, and other important signs.

*Surgery and pathology*: The patient underwent open-chest surgery on September 5, 2023. Intraoperatively, the RA was of normal size, containing a 3.0-cm × 5.0-cm mass with a smooth contour, broad-based pedicle, and minimal mobility. The neoplasm infiltrated the anterior/posterior RA walls, the superior vena cava (SVC) orifice, and the interatrial sulcus, with marked fibroedematous thickening of the involved tissues. The boundary between the tumor and the right atrial appendage and SVC ostium was ill-defined. The sinoatrial (SA) nodal complex and the surrounding adipose tissue were fibrosed and invaded. The tricuspid annulus was of normal size with trace regurgitation.

**Figure 2:**
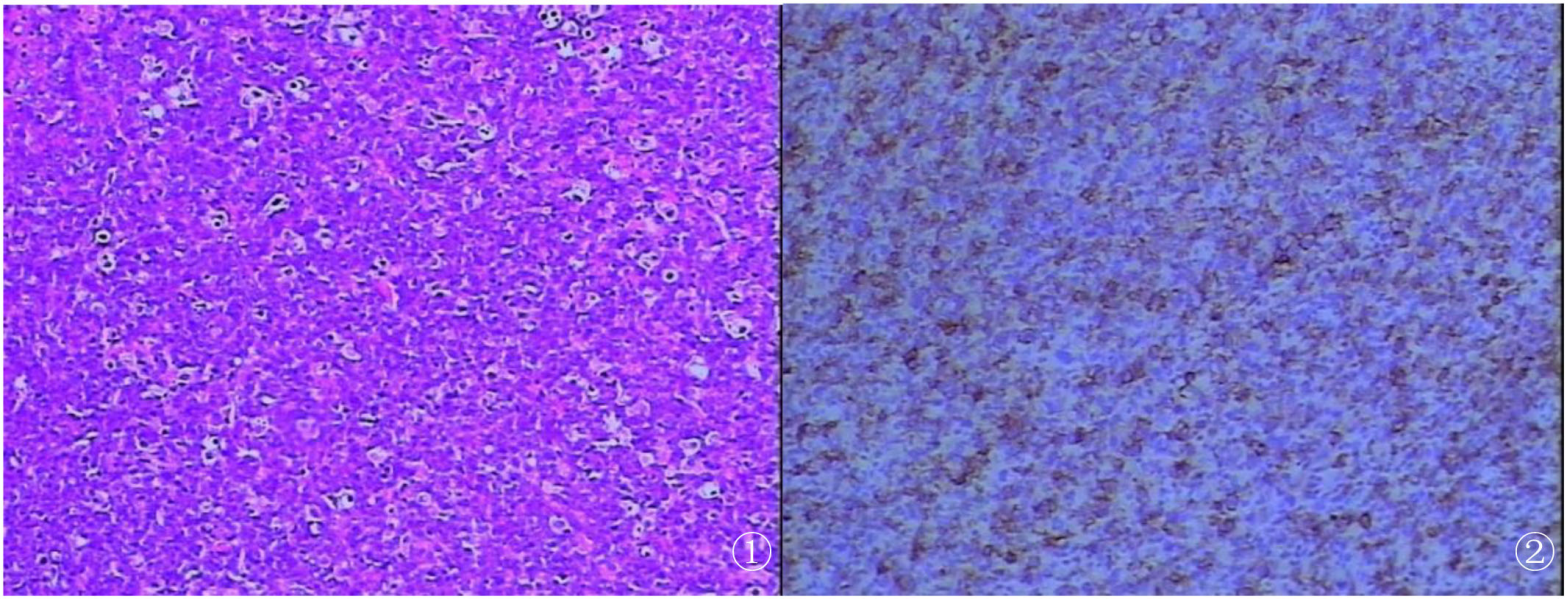
**(A)** Histological examination demonstrating diffuse proliferation of abnormal large lymphoid cells (hematoxylin and eosin stain). **(B)** Positive immunohistochemical staining of CD20 (B-cell marker) consistent with B-cell lymphoma (EnVision).

Pathological examination showed a 6.0-cm × 6.0-cm × 2.5-cm mass from the RA, partially encapsulated, with a tan-red to tan-white, solid, soft, and lobulated cut surface. The margin of the mass had a relatively intact capsule with focal areas of infiltration into adjacent tissues. Microscopy revealed diffuse sheets of atypical lymphocytes with a high nuclear-to-cytoplasmic ratio, large-to-medium pleomorphic cells with round to irregular nuclei, open chromatin, prominent nucleoli, and frequent mitotic figures (up to 20/10 high-power fields) (**Figure 2A**). The cytoplasm was scant to moderate, eosinophilic, and indistinct at the borders. A fibroedematous background was present within the mass, scattered with small lymphocytes (**Figure 2B**). Immunohistochemical staining showed diffuse positivity for CD20 and CD79a and focal positivity for MUM1 (50%+), C-myc (40%+), and BCL-2 (90%). The Ki-67 proliferation index was approximately 90%. The T-cell markers (CD3 and CD5), germinal center-associated markers (CD10 and BCL-6), epithelial markers (CK and EMA), EBV (Epstein–Barr virus-encoded small RNA, EBER), and immature lymphoid markers (CD99 and CD34) were negative. Clonal gene rearrangement studies of the IGH, IGλ, and IGλ genes were performed with polymerase chain reaction (PCR).

### Follow up

During the re-examination 2 months after surgery, the patient was found to have cardiac enlargement and pericardial effusion, with no recurrence in the surgical area (**Figure 1I**). However, the patient’s cardiac function showed progressive deterioration.

## Discussion

According to literature reports, the main clinical features of PCL included dyspnea, heart failure, precordial pain, life-threatening arrhythmias (due to conduction system invasion or myocardial irritation) and pleural/pericardial effusion, cardiac tamponade, or shock from outflow obstruction (4). The patient initially presented with dizziness, progressing to lower limb edema as the disease advanced.

### Comparative analysis with previous studies

Most reported cases of PCL show hypervascularity on first-pass perfusion imaging (5). However, in our case, the mass presented with homogeneous progressive enhancement, which is an atypical finding. This difference may be related to the microvascular structure and cellular composition of the tumor. The fibroedematous background observed in the pathological results may have affected the distribution and enhancement pattern of the contrast agent, resulting in this unique imaging feature. Further research on the correlation between pathology and imaging in PCL is needed to better understand such differences.

### Analysis of misdiagnosis causes

The initial misdiagnosis of this case as cardiac myxoma can be attributed to several factors. Firstly, echocardiography has limitations in accurately differentiating tumors with similar morphological features. The lack of blood flow signals in the mass on color Doppler was not specific enough to rule out myxoma, as some myxomas may also have sparse blood supply. Secondly, the lack of awareness of the imaging features of PCL among clinicians led to insufficient consideration of this rare disease. In addition, the relatively nonspecific symptoms of the patient also contributed to the difficulty of early diagnosis. This emphasizes the importance of comprehensive imaging evaluation, in particular MRI, in differentiating primary cardiac tumors.

### Prognosis-related discussion

The patient in this case died 8 months after surgery, despite initial symptom improvement. Surgical resection alone may not be sufficient for PCL, as it is a highly aggressive tumor. Chemotherapy is considered the cornerstone of treatment for PCL. The patient’s refusal of further chemotherapy likely had a significant negative impact on the prognosis. Studies have shown that combined chemotherapy regimens can improve the survival rate of patients with PCL. This case highlights the need for clinicians to fully communicate the importance of chemotherapy with patients and explore more effective treatment strategies, such as the combination of surgery, chemotherapy, and targeted therapy, to improve the prognosis of patients with PCL.

### Differential diagnosis

Cardiac myxoma, angiosarcoma, and malignant lymphoma are among the most common primary neoplasms of the heart in adults (6). The patient in this case was misdiagnosed as myxoma, the most common benign tumor of the atrium. The myxoma originated from the atrial septal fossa ovale with a pedicled, irregular shape, calcification, and bleeding. It showed high signal on T2WI, low perfusion on perfusion imaging, and obvious enhancement on LGE.

Angiosarcoma of the heart occurs mostly in the right atrium (>90%), with slightly high signal on T2WI, no enhancement in first-pass perfusion, but obvious enhancement in the delayed phase. Internal bleeding and necrosis of the mass can form polymorphic “cauliflora” changes, and “solar ray” manifestations can occur when the mass invades the pericardium (7).

### Value of MRI in PCL diagnosis and treatment

As demonstrated in this case, MRI provides detailed information about the tumor’s location, size, relationship with adjacent structures, and tissue characteristics. The ability to show the invasion of the right atrial wall and IVC inlet, as well as the unique enhancement patterns, helps in the differentiation of PCL from other cardiac tumors, reducing the misdiagnosis rate. Moreover, MRI can guide the selection of biopsy sites, ensuring that representative tumor tissues are obtained for accurate pathological diagnosis. In the treatment planning stage (8), MRI can also be used to evaluate the response to chemotherapy (9), providing valuable information for adjustment of treatment strategies.

## Data Availability

The original contributions presented in the study are included in the article/supplementary material. Further inquiries can be directed to the corresponding author.
